# A Four-Dimensional Model of Attention-Deficit/Hyperactivity Disorder: Toward Improved Recognition of Female ADHD

**DOI:** 10.3390/ijms27156748

**Published:** 2026-07-28

**Authors:** Jaroslaw Jozwiak

**Affiliations:** ALMA Therapy Center, Olesińska 21, 02-548 Warszawa, Poland; jaroslaw.jozwiak@centrumalma.pl

**Keywords:** ADHD, dopamine, tonic dopamine, phasic dopamine, emotional dysregulation, hyperfocus, procrastination, hypoactivity, alexithymia, sex differences, biomarker, precision psychiatry

## Abstract

Attention-deficit/hyperactivity disorder (ADHD) is traditionally defined by symptoms of inattention, hyperactivity, and impulsivity. However, growing evidence suggests that this framework incompletely reflects the underlying neurobiology and clinical heterogeneity of the disorder, particularly in females. Current models of dopaminergic neurotransmission propose that ADHD is characterized not by simple dopamine deficiency but by dysregulation of tonic and phasic dopamine signaling. Theoretical models propose that reduced tonic dopamine activity may coexist with enhanced stimulus-dependent phasic responses in ADHD. Whether such signaling differences produce alternating states of underactivation and hyperactivation within the same individual has not been directly demonstrated and constitutes a central hypothesis of the present framework. In this narrative hypothesis paper, I examine evidence linking dopaminergic dysregulation to a broader ADHD phenotype encompassing four functional domains: energy regulation, attention allocation, behavioral activation, and emotional reactivity and awareness. Within these domains, hyperactivity and hypoactivity, inattention and hyperfocus, impulsive responding and difficulty initiating action, and emotional hyperreactivity and alexithymia are treated as potentially related manifestations rather than established psychometric opposites. Neuroimaging studies report alterations in dopamine transporter and receptor availability, while clinical and behavioral studies document associations between ADHD and different, sometimes contrasting manifestations within the proposed domains. Importantly, internally experienced manifestations such as emotional dysregulation, hypoactivity, procrastination, and hyperfocus appear particularly relevant to female presentations of ADHD. I propose that ADHD should be conceptualized as a multidimensional disorder of dopaminergic regulation. This framework may better explain phenotypic diversity, improve identification of underrecognized presentations, and guide future biomarker-driven and precision-medicine approaches to ADHD.

## 1. Introduction

Attention-Deficit/Hyperactivity Disorder (ADHD) is a chronic, debilitating neurodevelopmental condition whose global prevalence is estimated at 8% in children [1] and 2.5% in adults [2]. However, current ADHD definition is historically rooted in a male-centric, childhood-focused model, creating a diagnostic gap where boys are identified 2–3 times more frequently than girls [1]. This disparity is unlikely to be fully explained by common-variant genetic differences alone. Indeed, genetic correlation analyses suggest near complete sharing of common variant effects across sexes and do not explain such sex bias [3]. Moreover, symptoms such as hyperfocus [4], procrastination [5], anergy [6] or emotional dysregulation [7] are neglected and left out of the clinical picture.

Because the classical symptomatology of ADHD only partially captures the true nature of the condition—particularly its core features of emotional and self-regulatory dysfunction—there remains a substantial gap between diagnostic descriptions and lived experience. As a result, self-advocates play a crucial role in disseminating knowledge about ADHD. By articulating the everyday realities of the disorder (differing from the diagnostic criteria), they help translate clinical constructs into meaningful, experience-based narratives, thereby significantly advancing public and professional understanding. Their valued input further highlights the gap between diagnostic criteria and lived clinical experience.

As emerging evidence suggests, the clinical phenotype of ADHD in females is characterized more prominently by internalizing, low-activation, and emotion-driven dysfunctions [8,9], whereas the current diagnostic frameworks remain disproportionately weighted toward externally observable manifestations such as hyperactivity and overt impulsivity. These internally experienced, regulation-related symptoms—often less disruptive to the environment but highly impairing at the individual level—appear to be more prevalent and clinically salient in women than in men with ADHD. This mismatch between the phenomenology of ADHD in women and the existing nosological definition likely contributes to delayed diagnosis, misclassification, and inadequate access to appropriate treatment in this population, with grave consequences [10].

Nevertheless, much of the evidence reviewed in this paper concerns ADHD populations generally and was not derived from direct, adequately powered sex-stratified comparisons. Therefore, the relevance of the proposed domains to female ADHD should be regarded as a clinically motivated hypothesis rather than an established female-specific model.

The cited clinical studies establish associations between ADHD and individual phenomena such as hypoactivity, hyperfocus, procrastination, emotional dysregulation, and alexithymia. They do not establish that these manifestations constitute opposite poles of continuous dimensions, occur alternately within the same individuals, or result from tonic–phasic dopamine dysregulation. Those propositions represent the interpretative and hypothesis-generating components of the present model. Accordingly, this paper distinguishes among three levels of inference: empirical observations reported in the cited literature, their integration within the proposed conceptual framework, and mechanistic predictions that require prospective testing.

In this framework, the term “dimension” refers to a functional domain of regulation rather than to a psychometrically established bipolar continuum. The paired manifestations within each domain are clinically contrasting but are not assumed to be mutually exclusive, inversely correlated, or opposite endpoints of a single latent variable. They may alternate across contexts, coexist within the same individual, or vary independently. Establishing their dimensional structure and within-person relationships is an empirical objective generated by the model rather than an assumption established by the literature reviewed here.

### Literature-Selection Approach

This article presents a narrative, hypothesis-generating synthesis rather than a systematic review. Relevant publications were identified through non-systematic, targeted literature searches and examination of references in pertinent reviews and primary studies in PubMed, conducted up to July 2026, supplemented by examination of reference lists from pertinent reviews and primary studies. Search terms included combinations of “ADHD” with “dopamine,” “tonic dopamine,” “phasic dopamine,” “hyperfocus,” “hypoactivity,” “fatigue,” “procrastination,” “emotional dysregulation,” “alexithymia,” “sex differences,” “dopamine receptor,” and “dopamine transporter.” Studies were selected for their relevance to the proposed clinical domains, potential biological mechanisms, sex-related presentation, or differential diagnosis. Priority was given to reviews, meta-analyses, large observational studies, neuroimaging or functional studies, and publications directly examining the manifestations discussed. Because study identification and selection did not follow a preregistered systematic-review protocol, the evidence synthesis may be subject to selection bias and should be interpreted as a conceptual integration intended to generate testable hypotheses rather than provide an exhaustive or quantitative assessment of the literature.

## 2. Tonic–Phasic Dopamine Signaling as a Proposed Mechanism

The tonic–phasic dopamine (DA) model provides one biologically plausible framework for interpreting the clinical manifestations discussed below, but it remains an indirect and unconfirmed explanation. The studies reviewed in this section do not demonstrate alternating hypo- and hyperdopaminergic states within individuals. Moreover, ADHD-related regulatory difficulties may involve interacting dopaminergic, noradrenergic, arousal-regulation, executive-control, and reward-processing mechanisms rather than a single neurotransmitter pathway.

In the framework proposed by Grace [11] and subsequently adapted to ADHD by Sikström and Söderlund [12], tonic and phasic dopamine signaling refer to distinguishable temporal modes of dopaminergic activity. Tonic signaling denotes the relatively sustained extracellular dopamine tone generated by ongoing neuronal activity and regulated partly by dopamine reuptake and autoreceptor feedback. Phasic signaling denotes transient, event-related increases in dopamine release associated with burst firing in response to salient, novel, rewarding, or behaviorally relevant stimuli. The ADHD-related hypothesis considered here is not simply that dopamine alternates globally between deficiency and excess. More specifically, it proposes that relatively reduced tonic signaling or altered tonic regulation may coexist with an increased ratio of stimulus-evoked phasic response to baseline under some conditions. Whether this pattern occurs in ADHD and predicts the proposed clinical manifestations has not been directly established.

If the tonic–phasic model accurately reflects dopaminergic dynamics in ADHD, differences in dopamine transporter and receptor expression and activity might be expected. However, the cited imaging findings are indirect and heterogeneous; they do not measure tonic-to-phasic transitions or establish the behavioral consequences proposed here. Indeed, neuroimaging studies have reported both increased and decreased dopamine transporter (DAT) and receptor (DR) availability across different brain regions, study populations, and imaging methodologies. Positron emission tomography study demonstrated that individuals with ADHD exhibit reduced availability of dopamine D2/D3 receptors and DAT in key regions of the mesoaccumbens pathway, including the nucleus accumbens and midbrain, with additional alterations observed in the caudate nucleus and hypothalamus [13]. Another study indicated reductions in both postsynaptic (D2/D3 receptors) and presynaptic (DAT) markers that co-localized within reward-related striatal circuits, which the authors interpreted as evidence of dysfunction across reward-related dopaminergic circuits rather than an isolated regional abnormality [14]. Other experimental data suggested region-specific alterations in dopaminergic signaling extending beyond the striatum, with evidence for receptor-level changes (e.g., reduced D5 receptor density in hippocampal regions) and broader dysregulation of dopamine-related circuitry implicated in cognitive and motivational processes [15,16].

Taken together, these studies establish abnormalities in dopamine-related markers in ADHD, although their direction and regional distribution are inconsistent. I interpret these findings as being compatible with—but not demonstrative of—variable dopaminergic signaling. The present model therefore hypothesizes that some contrasting behavioral and cognitive states may correspond to periods of relatively lower or higher dopaminergic signaling within an individual. Direct within-person neurochemical and behavioral studies are required to test this proposition.

For empirical purposes, the tonic–phasic hypothesis would require measurement of both components within the same individuals. Tonic signaling could be approximated by baseline indices of extracellular dopamine availability or dopaminergic activity obtained under standardized low-salience conditions. Phasic responsiveness could be operationalized as the within-person change from that baseline during experimentally controlled exposure to reward, novelty, urgency, or other salient events. These neurobiological measures should be collected alongside behavioral indices such as task-initiation latency, premature responding, persistence, and attentional disengagement. The hypothesis predicts that the magnitude of stimulus-evoked dopaminergic change relative to baseline will covary with context-dependent behavioral activation. It would be weakened if tonic and event-related measures were unrelated, if they failed to predict the proposed behavioral patterns, or if alternative measures of arousal, noradrenergic function, executive control, or reward processing accounted for the associations more adequately. Static differences in receptor or transporter availability alone cannot test this prediction.

### Relationship to Existing ADHD Models

The proposed framework overlaps with several established models of ADHD but differs in its emphasis. Arousal-regulation models describe difficulty maintaining an optimal activation state and may account for fluctuations between underactivation and excessive or poorly regulated activity [17]. Reward-sensitivity and delay-aversion models emphasize altered responses to reward magnitude, immediacy, and salience, providing an alternative or complementary explanation for impulsive engagement, task avoidance, and context-dependent hyperfocus [18]. Executive-dysfunction models focus on deficits in inhibition, working memory, task initiation, and goal-directed control and may explain the coexistence of procrastination with impulsive responding without requiring alternating dopaminergic states [19].

Cognitive disengagement syndrome, characterized by daydreaming, mental fogginess, slowed behavior, and reduced alertness, overlaps particularly with the proposed low-activation manifestations but is conceptually distinguishable from core ADHD symptoms and may represent a partly independent construct [20]. The present model is therefore not intended to replace these accounts. Rather, it organizes selected clinical manifestations across four regulatory domains and proposes activation threshold as one possible integrative mechanism. Comparative studies are required to determine whether this framework provides explanatory or predictive value beyond arousal, reward-processing, executive-function, and cognitive-disengagement models.

## 3. Translation into Symptom Development

In this paper, behavioral activation threshold is defined as the minimum degree of perceived salience, anticipated reward, novelty, urgency, or external prompting required for an individual to initiate and sustain goal-directed behavior. A relatively high threshold is operationally reflected by longer task-initiation latency, frequent failure to begin low-salience tasks, and disproportionate improvement when reward, urgency, or external structure is increased. A relatively low threshold is reflected by rapid initiation or capture of behavior in response to salient stimuli, including responses that occur before adequate evaluation or inhibition. The construct is behavioral and context-dependent; it is not equivalent to a directly measured neurochemical threshold. The proposed relationship between behavioral activation threshold and tonic–phasic dopamine signaling remains a mechanistic hypothesis.

Dopamine plays a pleiotropic role in the human brain. It is involved in motivation and behavioral activation, reward processing, executive functions, attention regulation, arousal, wakefulness and emotional regulation, to name a few. On this basis, I hypothesize that differences in tonic and stimulus-dependent phasic signaling may contribute to contrasting symptoms across these domains. This proposed connection is mechanistic interpretation rather than an inference directly established by the clinical studies reviewed below.

**Figure 1:**
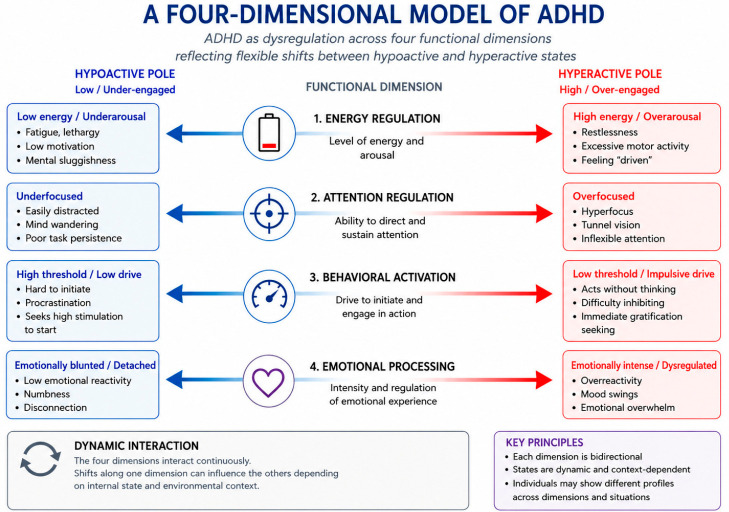
**The proposed four-dimensional model of ADHD.** Four-dimensional model of ADHD. In contrast to the traditional three-domain framework centered on inattention, hyperactivity, and impulsivity, the present model conceptualizes ADHD as dysregulation across four functional dimensions: energy regulation, attention regulation, behavioral activation, and emotional processing. Each dimension is provisionally represented as a continuum between two contrasting, clinically relevant manifestations. This arrangement is a hypothesis rather than an empirically established dimensional structure. The additional proposal that tonic–phasic dopaminergic dysregulation contributes to movement along these dimensions likewise requires direct testing.

The four domains (Figure 1) are defined by the regulatory function involved, not solely by the apparent opposition between their clinical manifestations. “Contrasting” therefore denotes a difference in observable or subjective presentation, not necessarily an inverse psychometric relationship. The conceptual basis and limitations of each proposed pairing are described below.

Hyperarousal/hyperactivity is one of the symptoms classically associated with ADHD [21]. Clinical studies separately associate ADHD with hyperactivity and, in a subset of patients, reduced activation, fatigue, or hypoactivity. The present framework interprets these phenomena as candidate manifestations of disturbed energy regulation. Their conceptual connection is based on their shared relationship to activity level and behavioral mobilization. However, the model does not assume that hyperactivity and hypoactivity are mutually exclusive endpoints of a single psychometric scale. They may occur in different contexts or at different times, and subjective exhaustion may coexist with observable restlessness. Their dimensional and temporal relationship, as well as any shared biological mechanism, requires direct investigation.

There is accumulating evidence that a subset of individuals with ADHD, particularly females, present with a contrasting phenotype marked by recurrent periods of reduced activation: chronic fatigue [22] and hypoactivity [6,23]. These low-energy states correlate well with inattention [8] which is—once again—more pronounced in females [24]. This hypoactive profile includes recurrent symptoms such as low initiative, lethargy, slowed cognitive tempo, and difficulty mobilizing effort, which are closely aligned with constructs such as sluggish cognitive tempo and anergia. Importantly, they are less likely to disrupt the external environment and therefore are less likely recognized as pathological, contributing to the underdiagnosis of ADHD in girls.

From the earliest clinical descriptions, disturbances in attention have been recognized as the central feature of what we now call ADHD. In his seminal lectures, Still (1902) described children who exhibited not only behavioral dysregulation but also marked difficulties in sustaining attention and maintaining goal-directed mental effort [21]. Also Kramer and Pollnow associated “hyperkinetic disorder” with attention problems [25]. Accumulating evidence led to a paradigm shift in DSM-III (1980), which explicitly redefined the disorder as Attention Deficit Disorder, thereby formalizing inattention as a core diagnostic domain.

Hyperfocus has also been reported more frequently in individuals with ADHD symptoms. Inattention and hyperfocus are not conceptualized here as opposite quantities of attention. Inattention refers to difficulty directing or sustaining attention according to current goals, whereas hyperfocus involves unusually persistent engagement with a selected activity and difficulty disengaging or shifting attention. Their proposed relationship therefore concerns dysregulated allocation and control of attention rather than opposite levels of attentional capacity. The two manifestations may coexist—for example, intense engagement with one activity may be accompanied by failure to attend to competing tasks or environmental information. Indeed, hyperfocus or “focus on details” [26] is often misinterpreted as a lack of ADHD, yet it appears to be a clinically meaningful and underrecognized feature of ADHD. Hyperfocus may be operationalized as a state of intense, sustained attentional engagement accompanied by characteristic features such as time distortion, diminished mindfulness of external stimuli, neglect of physiological needs, and marked difficulty disengaging or task-switching [27]. Within the proposed model, I hypothesize that hyperfocus may occur when a highly salient or intrinsically rewarding task exceeds an individual’s activation threshold. The further attribution of this transition to a tonic-to-phasic dopamine response is biologically plausible but has not been directly tested. Highly salient or intrinsically rewarding tasks may facilitate sustained engagement and make attentional disengagement more difficult [14]. Within the proposed framework, this pattern may reflect altered salience allocation and reward processing. A tonic-to-phasic dopamine response is one possible mechanism, but the cited evidence does not directly link such neurochemical transitions to episodes of hyperfocus; noradrenergic arousal and executive-control processes may also contribute.

Hupfeld conducted one of the first investigations on hyperfocus in adult ADHD using a newly developed, multidimensional instrument, the Adult Hyperfocus Questionnaire, validated across a pilot sample including ADHD cases [27]. ADHD symptom severity showed consistent, moderate associations with global hyperfocus scores and all domain-specific subscales, including school, hobbies, and screen-based activities. Individuals meeting ADHD criteria (self-report corroborated by Adult ADHD Self-Report Scale-Screener ≥ 14) exhibited significantly higher hyperfocus across all dimensions. Hyperfocus remained specifically linked to ADHD symptoms, independently of anxiety or depression. Importantly, factor analytic results supported hyperfocus as a multidimensional construct with distinct contextual and phenomenological components, while regression analyses identified scenario-based hyperfocus as the strongest predictor of ADHD status. These findings support an association between ADHD symptoms and context-dependent hyperfocus. The present model groups them within the domain of attention regulation because both may involve difficulty allocating and shifting attention in accordance with current goals. Psychometric and longitudinal studies are needed to determine whether they correlate, alternate across contexts, or constitute partly independent manifestations.

Importantly, patient experiences support the inclusion of hyperfocus within the ADHD phenotype. In a controlled study of adult patients with ADHD, hyperfocus severity was significantly higher compared to healthy controls (*p* < 0.001), with no difference between stimulant-naïve and stimulant-treated ADHD individuals, indicating that hyperfocus is not a medication-induced effect but rather an intrinsic feature of the disorder [28]. Furthermore, hyperfocus scores showed robust positive correlations with total ADHD symptom severity as measured by standardized instruments, including both inattention and hyperactivity/impulsivity domains, reinforcing its integration within the core psychopathological structure of ADHD. The presence of hyperfocus in ADHD is not merely epiphenomenal but clinically meaningful. Its association with functional impairment—such as neglect of responsibilities, social disruptions, and impaired task-switching—parallels that of typical ADHD symptoms.

Classically, impulsivity constitutes another core symptom dimension of ADHD and is conceptualized as a primary deficit in behavioral inhibition, which underlies broader impairments in self-regulation. In his seminal model, Russell A. Barkley posits that ADHD fundamentally reflects a deficit in the capacity to inhibit or interrupt responses and protect goal-directed behavior from interference [29] which may manifest clinically as a reduced threshold for response execution. The meta-analysis by Willcutt et al. demonstrated that individuals with ADHD exhibit significant impairments across all examined domains of executive functioning [30]. Notably, the most robust and consistent deficits were observed in the context of response inhibition, a core executive function directly underlying impulsivity. Importantly, these impairments were evident in both clinical and community samples and remained significant after controlling for intelligence, academic performance, and comorbid conditions. These deficits are consistently observed in laboratory paradigms, including go/no-go and stop-signal tasks, where individuals with ADHD demonstrate increased commission errors and impaired response inhibition [31]. Within the present framework, these findings are consistent with the hypothesis that highly salient stimuli facilitate rapid transitions into phasic dopaminergic signaling.

Procrastination is also frequently associated with ADHD [32,33,34]. Existing studies establish this association but do not show that procrastination is the opposite pole of impulsivity or that either phenomenon is determined by tonic dopamine signaling. Within the present framework, I hypothesize that impulsive responding and difficulty initiating low-salience tasks may represent contrasting manifestations of dysregulated behavioral activation.

Although procrastination may represent one behavioral manifestation of elevated activation threshold, it is not included among the formal diagnostic criteria for ADHD in DSM or ICD-based classifications. However, in a reliability and validity study on proposed DSM-5 ADHD symptoms by the DSM-5 Work Group, the symptom “reluctant to engage in “mental” tasks” ranked second among the 22 proposed symptoms, with 8 appearances among the 10 top-ranked three-item subsets in symptom-versus-impairment test [35]. The symptom has been incorporated into widely used screening instruments such as the Adult ADHD Self-Report Scale (ASRS), which includes an item directly capturing procrastination-related behavior (“How often do you avoid or delay getting started on tasks that require a lot of thought?”), reflecting its clinical salience in adults with ADHD. This inclusion is supported by evidence indicating that procrastination is highly prevalent in ADHD and closely linked to core symptom dimensions, particularly inattention and executive dysfunction. In a study performed on 2 thousand Hungarian students aged 18–35 years procrastination was most severely associated with combined type of ADHD, just above inattentive type. However, student recruitment procedure for the study excluded individuals who “reported difficulty understanding the questions” which might have affected the composition of the inattentive sample [36].

The study by Netzer Turgeman and Pollak investigated the mechanisms linking adult ADHD symptoms—particularly attention deficits—with procrastination in a large sample, using structural equation modeling to test motivational and emotional pathways [5]. The authors found a strong positive association between inattention and procrastination. This finding supports procrastination as a behavioral correlate of ADHD symptoms but does not directly implicate understimulation or tonic–phasic dopamine signaling. I interpret the association as compatible with the proposed activation-threshold model, while recognizing that alternative executive, motivational, and emotional mechanisms remain possible. Although ADHD symptoms were also associated with broader deficits in emotion regulation and goal-directed behavior, these factors did not independently account for procrastination when modeled simultaneously.

## 4. The Missing Piece…

Although emotional dysregulation (ED) was often associated with ADHD [37,38,39] and in their review Shaw et al. (2014) found that the phenomenon affects a high proportion (up to 70%) of ADHD individuals, both children and adults [40], it has never been included as one of the criteria for the diagnosis. While the diagnostic criteria for ADHD emphasize core symptoms of inattention and hyperactivity, ED provides a critical lens for mechanistically understanding the broad impairment profile and developmental trajectory of the disorder that may be sufficiently specific for ADHD to become one of the criteria [41], as it is significantly associated with ADHD, even when adjusting for other mental health conditions [42]. Empirically, the clinical relevance of this manifestation is substantial; children with ADHD are ten times more likely to demonstrate symptoms of ED than their typically developing peers [40,43], and they face a six-fold increased risk for displaying clinically significant emotional lability [44]. Importantly, adding ED to a regression model with the core symptoms of ADHD improved predictability of ADHD diagnosis [45].

Accumulating evidence suggests that emotional dysregulation constitutes a central dimension of the ADHD phenotype. In a well-characterized clinical sample of 213 medication-naïve adults with ADHD, confirmatory factor analysis demonstrated that ED, alongside positive and negative affect and emotion regulation skills, forms a distinct but closely interrelated domain with the classical symptom clusters of inattention, hyperactivity, and impulsivity [46]. Importantly, latent factor associations were especially strong between ED and impairments in self-concept, as well as with impulsivity/emotional lability, suggesting that emotional processes are deeply embedded within the core psychopathological architecture of ADHD. According to a review by Soler-Gutierrez (2023), it is not possible to establish a clear distinction between ADHD subtypes based on ED [7]. However, it has been observed that the combined subtype tends to be associated with higher ED and that the greater the severity of ADHD symptoms, the greater ED. 

Uncovering the symptom “gap” between boys and girls necessitates a longitudinal lens to observe how symptoms evolve through the transition into adolescence. Cross-sectional snapshots often fail to capture the hidden divergence in symptom trajectories. To address this, De Ronda et al. (2024) utilized a substantial sample of 417 participants (ages 8–18), including a critical longitudinal subsample of 121 participants providing multiple time-point data [9]. The longitudinal data revealed that for girls with ADHD, emotional symptoms represent a persistent, and in some cases worsening, burden throughout development. Unlike their male counterparts, girls with ADHD do not experience a “growth out of” pattern, suggesting that the female ADHD phenotype is characterized by a stable and entrenched dysregulation that resists the typical maturation process. Interestingly, there seems to be an overlap between ED and internalizing behavior. In a recent study on 1539 adults ED significantly improved the model for identification of impairment and internalizing problems, typical especially for females with ADHD [47]. ED is not only significantly associated with ADHD in women, but this group shows more frequent use of non-adaptive emotion regulation strategies [48].

## 5. …And Its Counterpart

Certainly, we lack a consistent definition of ED which makes comparison of different studies difficult. But if we think of ED as a transdiagnostic phenomenon [42,49], we can cite the classic definition of Linehan [50] describing ED as high emotional sensitivity, high emotional reactivity and slow return to baseline. What these three elements have in common is emotional hyperreactivity or strong perception of one’s feelings. Emotional dysregulation and alexithymia have each been associated with ADHD, but the available studies do not demonstrate that they form opposite poles of a single continuum. The present framework proposes this organization as a testable hypothesis: emotional processing may vary from rapid, intense emotional reactivity to reduced identification and differentiation of emotional states. These manifestations may also coexist or vary independently, an alternative that should be evaluated empirically.

Sifneos (1973) described for the first time alexithymia as impairments in the identification, differentiation, and verbalization of one’s own emotions, accompanied by a limited capacity for symbolic thinking (poor fantasy life) and a tendency toward externally oriented cognitive style [51]. Individuals with alexithymia have difficulty recognizing internal emotional states, distinguishing emotions from bodily sensations, and describing feelings to others. In a controlled pilot study, Donfrancesco examined alexithymia in a clinical sample of 50 stimulant-naïve children with ADHD compared with 100 age- and sex-matched typically developing controls [52]. Children with ADHD demonstrated significantly higher total alexithymia scores than controls, with the largest differences observed in difficulty identifying feelings and externally oriented thinking. Notably, alexithymia showed a moderate association with hyperactivity/impulsivity (r = 0.46, *p* = 0.001), whereas no significant relationship was observed with inattentive symptoms. Also on the sample of 101 adult ADHD patients, alexithymia correlated with impulsivity [53].

These findings indicate that alexithymia in ADHD is not a generalized deficit in emotional processing but is specifically linked to the behavioral dysregulation dimension of the disorder. Association with hyperactivity/impulsivity indicates a link between impaired emotional awareness and behavioral dysregulation. Whether impaired awareness contributes causally to impulsive behavior, results from it, or reflects shared underlying factors remains uncertain.

The manifestations incorporated into the proposed model are not specific to ADHD. Fatigue, reduced motivation, procrastination, and emotional blunting may occur in depressive and anxiety disorders, sleep disorders, medication-related states, and other medical or psychiatric conditions. Alexithymia is likewise transdiagnostic and is frequently examined in autism spectrum and other neurodevelopmental conditions, while low activation, mental fatigue, and slowed or disengaged cognition overlap with cognitive disengagement syndrome. Consequently, the presence of any individual manifestation should not be interpreted as evidence of ADHD or used as an independent diagnostic criterion.

### Transdiagnostic Overlap

Within the proposed framework, the potential relevance of these manifestations to ADHD depends on their relationship to the broader developmental pattern of ADHD symptoms, including difficulties with attention regulation, behavioral inhibition, task initiation, and context-dependent response to salience or reward. Differential assessment should consider developmental onset, persistence across settings, temporal relationships with mood and sleep disturbances, co-occurring conditions, medication effects, and whether the manifestation is better explained by another disorder. Future comparative studies should determine whether the proposed combinations, temporal patterns, or context dependence provide greater diagnostic specificity than the individual manifestations themselves.

## 6. Dopaminergic Receptor Variants and Individual Differences in ADHD Phenotypes

The genetic and molecular findings reviewed below establish effects of certain variants on receptor or transporter expression and function. They do not establish that these variants determine an individual’s tonic-to-phasic activation threshold, position on the proposed symptom dimensions, or transitions between contrasting states. Those links are hypotheses associated with the present model.

Contemporary genome-wide studies indicate that ADHD is highly polygenic, with risk distributed across numerous common variants of individually small effect [54]. Earlier candidate-gene studies reported associations involving dopaminergic genes, including *DRD2*, *DRD4*, *DRD5*, and *DAT1*; however, these findings have generally been modest, heterogeneous, and inconsistently replicated [55,56,57]. Functional effects of particular variants on receptor expression or signaling may establish biological plausibility, but they do not demonstrate that these variants explain ADHD or determine the clinical phenotypes proposed in this model. The following discussion should therefore be interpreted as hypothesis-generating rather than as evidence for variant-specific ADHD subtypes. Some of these variants influence receptor structure, whereas others alter receptor expression, splicing, intracellular signaling, or transcriptional regulation, and may thereby influence the physiological threshold required to transition from tonic to phasic signaling and/or affect signal duration.

Among the candidate variants discussed in relation to dopaminergic function, *DRD4* has been investigated in functional studies; however, evidence connecting individual *DRD4* variants to ADHD risk or to specific clinical phenotypes remains limited and inconsistent. Variable number tandem repeat (VNTR) polymorphisms in exon III of *DRD4* alter the structure of the third intracellular loop of the D4 receptor, a region critically involved in G-protein coupling and intracellular signal transduction [58]. For example, functional studies have demonstrated that the ADHD-associated D4.7 receptor exhibits reduced efficiency of dopamine-mediated intracellular signaling compared with shorter receptor variants, producing weaker inhibition of cAMP formation despite equivalent dopaminergic stimulation [58,59]. Consequently, higher extracellular dopamine concentrations may be required to achieve the same postsynaptic effect.

More recent evidence suggests that the functional consequences of *DRD4* polymorphisms extend beyond simple receptor sensitivity. The D4.4 and D4.7 receptor variants differ in their ability to form heteromeric complexes with D2 and α2A receptors, thereby modifying dopaminergic and noradrenergic regulation of cortico-striatal circuits involved in attention, motivation, and executive control [58]. Such differences may alter the amplification of dopaminergic signals reaching frontal cortical networks. Similar mechanisms may also operate through *DRD2*. Several *DRD2* polymorphisms have been proposed to influence receptor expression, mRNA stability, alternative splicing, receptor density, and intracellular signaling efficiency [57,60,61,62,63]. Although the evidence linking specific *DRD2* variants to ADHD is less consistent than for *DRD4*, these findings support the broader hypothesis that genetic variation can modify the biological response to dopamine rather than dopamine availability itself. The present model hypothesizes that variant-related differences in receptor expression or signaling efficiency could modify responsiveness to dopaminergic stimulation [60,64]. Whether these molecular differences alter tonic-to-phasic transitions or explain individual positions on the proposed symptom dimensions (Table 1) remains unknown.

## 7. Dopamine Transporter Polymorphisms as Modulators of Dopaminergic Signaling in ADHD

The dopamine transporter (DAT), encoded by the *SLC6A3* (*DAT1*) gene, is the principal mechanism responsible for terminating dopaminergic neurotransmission through reuptake of dopamine from the synaptic cleft. Consequently, genetic variants affecting DAT expression or function have the potential to alter extracellular dopamine availability and the temporal dynamics of dopaminergic signaling [65]. This biological role has made *DAT1* one of the most extensively investigated candidate genes in ADHD.

Evidence linking *DAT1* polymorphisms to ADHD emerged from early family-based association studies. Cook et al. [65] first reported preferential transmission of the 10-repeat (10R) allele of the 40 bp VNTR located in the 3′ untranslated region of *DAT1* to children with ADHD. Subsequent studies replicated this observation in independent cohorts [66,67]. In a family-based association study involving UK and Turkish samples, Curran et al. [68] found significant over-transmission of the 10R allele in the UK cohort, although the finding was not replicated in the Turkish sample, suggesting genetic heterogeneity across populations. Importantly, when the authors combined all available family-based studies, they observed modest overall evidence for association and concluded that variation at the *DAT1* locus may contribute to ADHD susceptibility, albeit with small effect sizes. Several studies have demonstrated functional consequences of *DAT1* polymorphisms. Fuke et al. [69] showed that the VNTR polymorphism influences *DAT1* gene expression, indicating that genetic variation may directly alter transporter abundance. Similarly, other authors demonstrated that the 3′-UTR VNTR regulates *DAT1* expression levels in human tissues [70]. These findings indicate that some *DAT1* variants can affect gene expression in experimental or selected biological contexts. However, functional effects on transporter expression do not establish that these variants have clinically meaningful effects on ADHD risk or account for the proposed activation-threshold phenotypes.

Further support for the functional significance of *DAT1* variation comes from neuroimaging studies reporting that individuals carrying different *DAT1* genotypes exhibited differences in striatal dopamine transporter availability in vivo [71]. Specifically, carriers of the 9R/10R genotype showed approximately 22% lower DAT availability in the putamen compared with 10R homozygotes, indicating that *DAT1* polymorphisms may influence transporter density in the human brain. Consistent with this observation, imaging studies in adults with ADHD demonstrated increased striatal DAT density relative to controls, suggesting that altered dopamine transporter regulation may contribute to ADHD pathophysiology [72,73].

Additional evidence was provided by Pinsonneault et al. [74], who identified regulatory *DAT1* variants capable of altering transporter expression in human brain tissue. Their findings established a direct molecular mechanism through which *DAT1* polymorphisms can influence dopamine clearance. Because DAT is the primary determinant of extracellular dopamine duration and amplitude [75], the available evidence suggests that *DAT1* polymorphisms contribute to ADHD not only through statistical genetic association but also through measurable effects on transporter expression and dopamine signaling [76,77]. Variants associated with increased *DAT* expression may accelerate dopamine reuptake, reducing the amplitude and duration of extracellular dopamine signals, whereas variants associated with reduced transporter activity may prolong dopaminergic transmission. These effects provide biological plausibility for the hypothesis that *DAT1* variation may influence dopaminergic responsiveness. However, the cited studies did not measure the proposed activation threshold or relate it directly to the four symptom dimensions.

Within the four-dimensional framework, receptor sensitivity and transporter availability are proposed as possible modifiers of dopaminergic responsiveness. In principle, variation affecting receptor or transporter function could influence the amplitude or duration of dopamine signaling. However, no specific *DRD2*, *DRD4*, *DRD5*, or *DAT1* variant has been shown to determine an individual’s activation threshold, position within the proposed clinical domains, or transitions between contrasting states. Given the highly polygenic architecture of ADHD, any such effects would probably be small and operate alongside numerous genetic and environmental influences. Direct genotype–phenotype and neurobiological studies are required to test this hypothesis.

## 8. Implications for the Clinical Picture

If individual genetic differences in dopamine (and possibly other inter- and intracellular pathways) underlie the threshold at which tonic activity is converted into phasic response as well as the duration of phasic response, ADHD may encompass clinically distinct presentations that differ in predisposition to specific regions on the four axes of the proposed model. Indeed, the MTA follow-up demonstrated marked longitudinal fluctuation of ADHD symptom severity in approximately two-thirds of participants, illustrating the dynamic nature of ADHD clinical expression [78]. The model predicts that one region of the proposed spectrum would include individuals who appear highly responsive to salient environmental stimuli and rapidly transition into states of behavioral activation (i.e., fulfill most of current criterial symptoms). Clinically, these patients often present with impulsivity, sensation seeking, reward-driven behavior, emotional lability, and difficulty delaying gratification, all of which have been repeatedly linked to ADHD [30,79,80]. They may become intensely engaged by novel opportunities, social interactions, hobbies, or immediate rewards, yet struggle to maintain stable goal-directed behavior because of the presence of competing stimuli that may shift their attention. Such individuals frequently report impulsive spending, excessive risk-taking, problematic substance use, unstable relationships, and rapid shifts of interest. Emotional responses may be intense and immediate, with frustration, anger, excitement, or rejection sensitivity escalating within seconds of exposure to emotionally salient events [40].

The model predicts that another region of the proposed spectrum would include individuals who appear to require substantially greater stimulation before entering a state of behavioral activation. Rather than exhibiting excessive impulsivity, they often struggle with task initiation, sustained effort (because of low tonic energy level), and motivational drive. Their clinical presentation is frequently characterized by chronic procrastination, difficulty starting tasks (even despite adequate intentions), low subjective energy, mental fatigue, and persistent underachievement relative to intellectual ability. Procrastination has consistently been associated with ADHD and is now recognized as one of the most common functional complaints reported by adults with the disorder [81,82]. Some individuals with ADHD report periods of exceptional productivity under urgency, novelty, competition, or emotional pressure [23]. Prospective within-person studies are needed to establish whether these periods occur preferentially in individuals with otherwise elevated activation thresholds and whether dopaminergic transitions account for this pattern.

These proposed patterns are not mutually exclusive clinical types. The same person may have difficulty initiating low-salience tasks while responding impulsively to immediate rewards, or may display inattention toward competing demands while remaining intensely engaged with one salient activity. The model therefore predicts context-dependent combinations of manifestations rather than consistent placement at one corresponding pole across all four domains.

Differences in activation threshold may contribute to heterogeneity in emotional reactivity, although this relationship remains hypothetical. Relatively lower behavioral activation threshold might be associated with rapid emotional responses, reward sensitivity, or emotionally driven behavior [82], whereas relatively higher activation threshold might be associated with difficulty mobilizing emotional or motivational engagement in some contexts. Emotional awareness constitutes a separate component: alexithymia may occur alongside either high or low observable emotional reactivity and should not be inferred from activation threshold alone. These profiles may overlap within individuals and should not be interpreted as mutually exclusive groups.

This four-dimensional model may further help explain why many individuals with predominantly inattentive presentations remain unrecognized for years, with substantial negative consequences [10,83,84]. While highly activated individuals often attract clinical attention because of disruptive, impulsive, or hyperactive behavior [85], those with chronically elevated activation thresholds may present with internalized symptoms, academic underperformance, procrastination, emotional distress, and low self-esteem despite substantial functional impairment [86]. Consequently, differences in dopaminergic activation dynamics may represent one potential mechanism underlying the remarkable clinical heterogeneity observed across ADHD populations [87].

## 9. Testable Predictions and Validation Strategy

The proposed model generates several predictions that can and should be evaluated empirically. First, psychometric studies should determine whether manifestations assigned to each regulatory domain form coherent latent constructs and whether their relationships are bipolar, positively associated, or partly independent. The model would be challenged if the proposed groupings showed no reproducible structure or were better explained entirely by existing ADHD symptom dimensions.

Second, longitudinal within-person studies using ecological momentary assessment, repeated behavioral tasks, or digital activity measures should test whether hypoactive and hyperactive states, inattention and hyperfocus, and delayed versus rapid behavioral activation vary systematically with context, salience, reward, novelty, or urgency. Such studies should also determine whether contrasting manifestations alternate within individuals or coexist during the same periods.

Third, adequately powered sex-stratified studies should examine whether the prevalence, severity, functional consequences, and diagnostic visibility of the proposed manifestations differ between females and males. The hypothesis that more internalized or higher-activation-threshold patterns contribute to underrecognition in females would be supported only if these patterns were more frequent or less likely to prompt diagnosis in females after accounting for overall ADHD severity and co-occurring conditions.

Fourth, multimodal neurobiological studies should test whether individual differences in the proposed activation threshold are associated with independently measured dopaminergic responsiveness. Ideally, neural or neurochemical measures should be obtained repeatedly under low- and high-salience conditions and related to simultaneous behavioral changes. Dopamine transporter or receptor availability measured at a single time point would not be sufficient to demonstrate tonic-to-phasic transitions. Noradrenergic activity, arousal regulation, executive control, and reward processing should be measured as competing or interacting mechanisms.

Fifth, prospective treatment studies should determine whether baseline dimensional profiles predict differential responses to pharmacological or non-pharmacological interventions. Any prediction should be tested in independent samples and should provide incremental value beyond established ADHD presentation, symptom severity, age, sex, and comorbidity. Until such predictive validity is demonstrated, the model should not guide treatment selection or stimulant dosing in clinical practice.

Behavioral activation threshold could be estimated experimentally by systematically varying task salience, reward magnitude, urgency, novelty, or external prompting and measuring task-initiation latency, response probability, persistence, and premature responding. Ecological momentary assessment could additionally examine how these contextual factors predict initiation and sustained engagement in everyday activities. A valid activation-threshold measure should demonstrate reliability, distinguish initiation difficulty from response-inhibition deficits, and predict behavior beyond conventional ADHD symptom severity.

## 10. Concluding Remarks

The current nosological model of ADHD may not fully capture clinically relevant phenomena reported by many individuals, particularly internally experienced manifestations that may contribute to underrecognition in women. Existing evidence supports associations between ADHD and hypoactivity, hyperfocus, procrastination, emotional dysregulation, and alexithymia. However, it does not establish that these manifestations form four bipolar continua or arise from tonic–phasic dopaminergic dysregulation. The four-dimensional model represents an interpretative synthesis and a set of testable hypotheses. It proposes that selected ADHD-related manifestations can be organized within four functional regulatory domains: energy regulation, attention regulation, behavioral activation and inhibition, and emotional reactivity and awareness. It does not assume that the manifestations within each domain are psychometric opposites. Rather, they may coexist, alternate across contexts, or vary independently. Differences in dopaminergic responsiveness are proposed as a possible contributor to these patterns. Psychometric analyses should determine whether the manifestations within each domain are inversely related, positively associated, or statistically independent, while longitudinal within-person studies should examine whether and under what conditions they alternate or coexist. Also, because these manifestations are transdiagnostic, they should prompt assessment of mood, anxiety, sleep, autism spectrum, cognitive disengagement, and other possible explanations rather than being interpreted as specific indicators of ADHD. Their incremental diagnostic value requires prospective comparative validation.

Importantly, the proposed framework is a hypothesis-generating research model and should not be interpreted as a clinically validated classification system. The four domains and the behavioral activation-threshold construct have not yet undergone psychometric, longitudinal, neurobiological, or predictive validation.

The proposed dopaminergic account should not be interpreted as an established or exclusive mechanism. Dopamine transporter and receptor findings provide evidence of dopamine-related abnormalities at the group level but do not demonstrate within-person alternation between hypo- and hyperdopaminergic states. Future studies should compare the explanatory value of tonic–phasic dopamine signaling with, and examine its interaction with, noradrenergic signaling, arousal regulation, executive control, and reward-processing pathways.

This expanded model may have implications for the recognition of presentations that are less externally observable. Girls and women with ADHD are frequently underrecognized, and internalizing or less disruptive presentations may contribute to delayed diagnosis. However, most manifestations discussed in this paper are not unique to females, and the available evidence does not establish that the proposed four-domain or activation-threshold profiles differ systematically by sex.

Beyond its implications for clinical phenotyping, the proposed four-dimensional model has important consequences for future ADHD research. If individual differences in dopaminergic activation threshold and duration contribute substantially to the clinical phenotype, then studies investigating genetics, neurobiology, neuroimaging, cognitive functioning, and treatment response should account for this rather than treating ADHD as a biologically homogeneous disorder. Failure to stratify participants according to activation-threshold phenotype may obscure biologically meaningful associations and partly explain the inconsistent or heterogeneous findings frequently reported in ADHD research [88,89]. The present model therefore provides a framework for a more biologically informed classification of ADHD that may facilitate biomarker discovery and precision psychiatry.

The model also generates hypotheses for future treatment research. Prospective studies should test whether baseline behavioral activation-threshold profiles predict differential responses to pharmacological or non-pharmacological interventions. Such studies could examine whether dimensional profiles are associated with variation in treatment efficacy, adverse effects, or dose–response relationships, including stimulant titration. These possibilities have not been prospectively validated and should not currently guide treatment selection, stimulant dosing, or other clinical decisions. If replicated in independent samples and shown to provide predictive value beyond established clinical factors, activation-threshold phenotyping could eventually contribute to treatment-personalization research.

## Figures and Tables

**Table 1:** Hypothesized clinical patterns associated with variation in behavioral activation threshold. Note: The phenotypes and their placement on a continuous spectrum are theoretical predictions. The cited evidence supports associations between ADHD and the individual clinical manifestations but does not establish their organization into opposite poles or their proposed tonic–phasic dopaminergic mechanism. Individual differences in dopaminergic signaling dynamics may influence the threshold at which tonic dopamine activity transitions into behaviorally relevant phasic signaling. Individuals with lower activation thresholds are hypothesized to shift more readily into phasic activation, resulting in a clinical presentation characterized by frequent hyperactivity, impulsivity, emotional hyperreactivity, and rapid engagement with salient stimuli. In contrast, individuals with higher activation thresholds may require substantially greater motivational stimulation before behavioral activation occurs, leading to a phenotype dominated by more frequent hypoactivity, procrastination, reduced initiative, and internalized symptoms. These proposed phenotypes are not intended to represent discrete ADHD subtypes but rather opposite ends of a continuous spectrum.

Characteristic	(Tendency to Display) Low Activation-Threshold Phenotype	(Tendency to Display) High Activation-Threshold Phenotype
**Proposed dopaminergic dynamics**	Rapid transition from tonic to phasic dopamine signaling	Greater stimulation required to trigger phasic dopamine signaling
**Behavioral activation**	Easily initiated	Difficult to initiate
**Dominant behavioral pattern**	Externalizing	Internalizing
**Energy profile**	Hyperactivity, restlessness	Hypoactivity, fatigue, low initiative
**Attention profile**	Easily captured by salient stimuli; frequent hyperfocus	Persistent inattention until sufficient stimulation occurs
**Activation threshold**	Impulsive reaction	Procrastination, delayed task initiation
**Emotional profile**	Emotional hyperreactivity, emotional lability	Emotional blunting or reduced emotional awareness (alexithymia)
**Reward processing**	Strong attraction to immediate rewards and novelty	Requires stronger reward value before behavioral engagement
**Typical functional difficulties**	Risk-taking, substance misuse, impulsive spending, unstable relationships	Underachievement, chronic procrastination, missed deadlines, low self-esteem
**Typical diagnostic outcome**	More likely to fulfill current DSM/ICD criteria early in life	More likely to remain underrecognized or receive delayed diagnosis

## Data Availability

No new data were created or analyzed in this study. Data sharing is not applicable to this article.
